# Supplementary material for: The function of Anr in the differential effects of oxygen levels on biofilm development and nitrogenase performance in Pseudomonas stutzeri A1501
Source: PLoS One. 2025 Sep 24;20(9):e0333183. doi: 10.1371/journal.pone.0333183 (PMC12459779; doi:10.1371/journal.pone.0333183)
Supplement: S1 Fig — (PDF) [file pone.0333183.s001.PDF]

Supplementary Fig S1

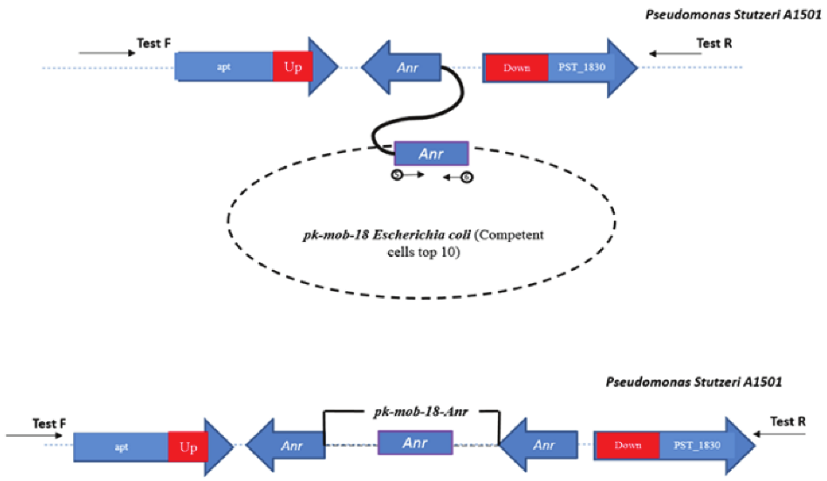

Supplementary Fig. S1. Illustration showing the pk-mob-18 plasmid with a defective *anr* insertion in the genome of *P. stutzeri* A1501.
