# Supplementary material for: The function of Anr in the differential effects of oxygen levels on biofilm development and nitrogenase performance in Pseudomonas stutzeri A1501
Source: PLoS One. 2025 Sep 24;20(9):e0333183. doi: 10.1371/journal.pone.0333183 (PMC12459779; doi:10.1371/journal.pone.0333183)
Supplement: S2 Fig — (PDF) [file pone.0333183.s002.PDF]

Supplementary Fig S2

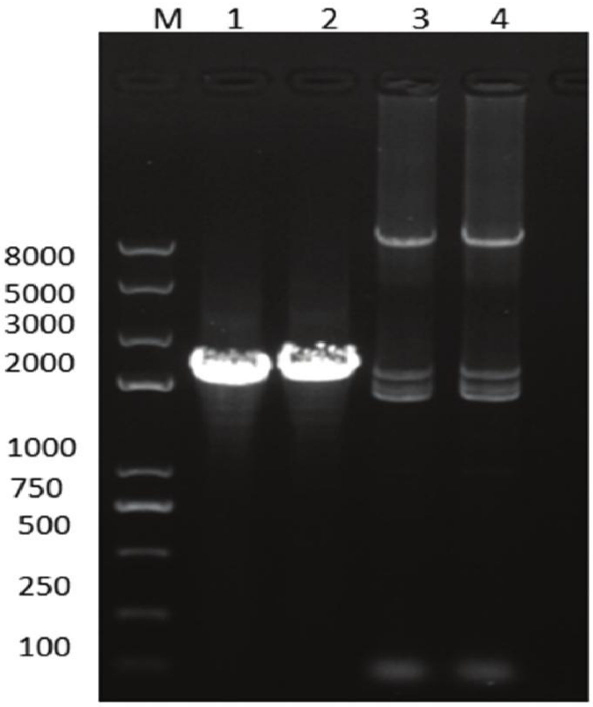

Supplementary Fig. S2. Gel electrophoresis bands 1-2 amplified *anr* (up and down expression genes) from *P. stutzeri* A1501 wild type showing a single band and 3-4 amplified with pk-mob-18 with multiple bands confirming insertion.
