# Supplementary material for: The function of Anr in the differential effects of oxygen levels on biofilm development and nitrogenase performance in Pseudomonas stutzeri A1501
Source: PLoS One. 2025 Sep 24;20(9):e0333183. doi: 10.1371/journal.pone.0333183 (PMC12459779; doi:10.1371/journal.pone.0333183)
Supplement: S4 Fig — Their growth represents no significant change in the growth after 10-fold serial dilution between Δanr and in wild type A1501. (PDF) [file pone.0333183.s004.PDF]

Supplementary Fig S4

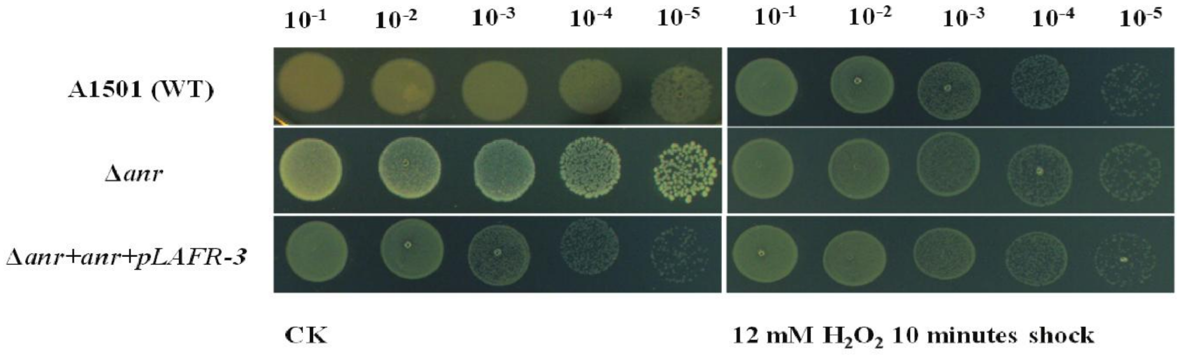

Supplementary Fig. S4. Oxidative stress test after exposure to 12mM of H2O2 for 10 minutes. Their growth represents no significant change in the growth after 10-fold serial dilution between Δanr and in wild type A1501.
