# Supplementary material for: The function of Anr in the differential effects of oxygen levels on biofilm development and nitrogenase performance in Pseudomonas stutzeri A1501
Source: PLoS One. 2025 Sep 24;20(9):e0333183. doi: 10.1371/journal.pone.0333183 (PMC12459779; doi:10.1371/journal.pone.0333183)
Supplement: S1 Table — (PDF) [file pone.0333183.s006.PDF]

Supplementary Table

Supplementary Table-S1. Reagents used

Description	Source	Identifier
Xba I	NEB	Cat# R0145S
Sph I-HF	NEB	Cat# R3182S
Nhe I-HF	NEB	Cat# R3131S
EcoR I-HF	NEB	Cat# R3101S
BamH I -HF	NEB	Cat# R3136S
Hind III-HF	NEB	Cat# R3104S
Nde I	NEB	Cat# R0111S
Spe I-HF	NEB	Cat# R3133S
Xho I	NEB	Cat# R0146S
Nco I-HF	NEB	Cat# R3193S
Yeast Extract Powder	Oxoid	Cat# LP0021B
DMSO	Invitrogen	Cat# D12345
Trypton	Oxoid	Cat# LP0042B
Ethanol	Thermo Scientific	Cat# 611050040
Ampicillin	Sigma-Aldrich	Cat# A5354
Gentamicin	Sigma-Aldrich	Cat# G1264-5G
Hygromycin B	Sigma-Aldrich	Cat# SBR00039
Kanamycin	Sigma-Aldrich	Cat# E004000-5G
Tetracycline hydrochloride	Thermo Scientific	Cat# 233100025
HEPES buffer	GIBCO	Cat# 15630080
Trimethylamine	Sigma-Aldrich	Cat# 471283
b-Mercaptoethanol	Sigma-Aldrich	Cat# 444203

DTT	Thermo Scientific	Cat# R0861
RNAlater solution	Invitrogen	Cat# AM7020
