# Supplementary material for: The function of Anr in the differential effects of oxygen levels on biofilm development and nitrogenase performance in Pseudomonas stutzeri A1501
Source: PLoS One. 2025 Sep 24;20(9):e0333183. doi: 10.1371/journal.pone.0333183 (PMC12459779; doi:10.1371/journal.pone.0333183)
Supplement: S2 Table — (PDF) [file pone.0333183.s007.PDF]

Supplementary Table S2: List of Primers used:

Primer Name	Primer sequence(5'to3')
**Anr-1-F**	CGGTCGACGATGGCGGTGAAGAACAGAT
**Anr-1-R**	CCGAATTCCTGCCAGCCCTAGGTAGTTG
**Anr-As-F**	GCTATGACCATGATTACGAATTCCTGCCAGCCCTAGGTAGTTG
**Anr-As-R**	AGCTTGCATGCCTGCAGGTCGACCGATGGCGGTGAAGAACAGAT
**pk-18-C-F**	TGCAGGCATGCAAGCTTGGCACTGG
**pk-18-C-R**	CCAGTGCCAAGCTTGCATGCCTGCA
**Anr-C-F**	GAGGATATTGGCCTGATCCA
**Anr-C-R**	AGAAACTGGCAATGCTGGAG
**Anr- F-PL**	ATCGGGATCCTGGCGCCGCCGGCGAGGGCGCACA
**Anr- R-PL**	ATCGAAGCTTCCGAATCGATCAAGGTCCGTGCGCA
**pLAFR – 3 Complimentary C –F:**	ACGTTGTAAAACGACGGCCAGTGCC
**pLAFR – 3 Complimentary C –R:**	CTATGACCATGATTACGAATTCCCG
**Anr –P-R**	TTAAGAAGGAGATATACCATGGCATCCGAATCGATCAAGGTCCGT
**Anr –P-F**	CCGCGTGGCACCAGAGCGAGCTCGACGTCCATGGCGCCGCCGGCG
**Anr Forward Primer pET52b**	TCCTCTTTCAGGGACCCGGGTACCAGGATCCATGTCCGAATCGATCAAGG
**Anr Reverse Primer pET52b**	GTGGCACCAGAGCGAGCTCTGCGGCCGCGTCGACTCAGACGTCCATGGCG
**RpoS Forward Primer pET52b**	GAAGTCCTCTTTCAGGGACCCGGGTACCAGGATCCATGGCACTTAAAGAC
**RpoS Reverse Primer pET52b**	CGTGGCACCAGAGCGAGCTCTGCGGCCGCGTCGACTCACTGGAACAGCGC
