# Supplementary material for: The function of Anr in the differential effects of oxygen levels on biofilm development and nitrogenase performance in Pseudomonas stutzeri A1501
Source: PLoS One. 2025 Sep 24;20(9):e0333183. doi: 10.1371/journal.pone.0333183 (PMC12459779; doi:10.1371/journal.pone.0333183)
Supplement: S3 Table — (PDF) [file pone.0333183.s008.PDF]

Supplementary Table S3: List of qPCR primers used:

Primer Name	Primer sequence(5'to3')
**RT16S-F**	CCTACGGGAGGCAGCAG
**RT16S-R**	ATTACCGCGGCTGCTGG
**RT-ntrC-F**	GATCAATGGCGAATCGGGTAC
**RT-ntrC-R**	CAGCTCGGATTCCATCAGGTC
**RT-nifA-F**	CGCGAAGACCTCTACTACCG
**RT-nifA-R**	CAGCTTGGTTTGCGACCCT
**RT-nifH-F**	GAGATGATGGCGATGTATGC
**RT-nifH-R**	GGTCGGTGTTGCGGCTGTTG
***gacS*-F qpcR**	CAGCCGTATCAAGTCCGAGT
***gacS*-R qpcR**	AACGGAATGGTATCCAGCAC
***gacA*-F qpcR**	CGAAGAAGATCCGTTCCCTA
***gacA*-R qpcR**	CTTCT*GACA*ATTGGCGATCA
**RpoS F-qPCR**	ATGGCACTTAAAGACCAAGCGCTGG
**RpoS R-qPCR**	TCACTGGAACAGCGCGTCGCTCGAC
**NarL F-qPCR**	ATGATGCGTCGGGGTCTGCGTGACC
**NarL R-qPCR**	TCAACCTTTCGCCTCGTTCTCCAGC
