# Supplementary material for: The function of Anr in the differential effects of oxygen levels on biofilm development and nitrogenase performance in Pseudomonas stutzeri A1501
Source: PLoS One. 2025 Sep 24;20(9):e0333183. doi: 10.1371/journal.pone.0333183 (PMC12459779; doi:10.1371/journal.pone.0333183)
Supplement: S4 Table — (PDF) [file pone.0333183.s009.PDF]

Supplementary Table S4: Software and Algorithms

Description	Source	Identifier
GraphPad Prism	https://www.graphpad.com/scientific-software/prism/	Version 9.0.0
Chimera	https://www.cgl.ucsf.edu/chimera/	Alpha version 1.14
Cluspro	https://cluspro.org/login.php	Kozakov et al., 2017
Expasy	https://web.expasy.org/translate/	Schwede et al., 2003
Adobe Illustrator	https://www.adobe.com/cn/products/illustrator.	Version 2020 24.0.1
Adobe Photoshop	https://www.adobe.com/cn/products/photoshop/	Version 21.0.2
Microsoft office		Microsoft office 2019
MEGA 7	https://www.megasoftware.net	Version 7
Snapgene 3.2.1	https://www.snapgene.com/	Version 3.2.1
