# Supplementary material for: The function of Anr in the differential effects of oxygen levels on biofilm development and nitrogenase performance in Pseudomonas stutzeri A1501
Source: PLoS One. 2025 Sep 24;20(9):e0333183. doi: 10.1371/journal.pone.0333183 (PMC12459779; doi:10.1371/journal.pone.0333183)
Supplement: S5 Table — (PDF) [file pone.0333183.s010.PDF]

Supplementary Table S5: Anr promoter with other genes from Virtual footprinter (https://www.prodoric.de/vfp/vfp_promoter.php):

Pos. (Strand)	Binding sequence	Score (Core Score)	Gene/Locus	Dist. to ATG	Product
693193 (+)	TTGTTATCGATCAA	14.59 -9.54	PST_RS03150	45	MBL fold metallo-hydrolase
766740 (+)	TTGACCTGCATCAG	14.4 -9.54	PST_RS03445	-94	hypothetical protein
994542 (+)	TTGACCTGAGTCAA	14.46 -9.54	PST_RS04570	-104	MFS transporter
1050569 (+)	TTGATTGGCATCAG	14.13 -9.54	PST_RS04825	-71	CopD family protein
1051460 (+)	TTGACGGCGATCAA	14.85 -9.54	*mscL* PST_RS04835	-161	large-conductance mechanosensitive channel protein MscL
1053163 (+)	TTGATCTGCATCAA	15.36 -9.54	PST_RS04845	-210	ATP-binding protein
1423319 (+)	TTGATCTGCATCAA	15.36 -9.54	*rsxA* PST_RS06705	-141	electron transport complex subunit RsxA
1637976 (+)	TTGCCTTGAGTCAA	13.9 -9.54	*pdxH* PST_RS07735	-132	pyridoxamine 5'-phosphate oxidase
1643477 (+)	TTGCTCGGCATCAA	14.59 -9.54	PST_RS07760	344	monovalent cation/H+ antiporter subunit D
1705991 (+)	TTGTCCTGGATCAG	13.89 -9.54	PST_RS08075	-258	HPF/RaiA family ribosome-associated protein
1707716 (+)	TTGACCTGGATCAG	14.31 -9.54	PST_RS08090	-197	methyl-accepting chemotaxis protein
1784825 (+)	TTGACGCAGATCAA	14.79 -9.54	PST_RS08435	-91	AraC family transcriptional regulator
1791106 (+)	TTGCTGGGCATCAA	14.58 -9.54	PST_RS08465	-182	muconate cycloisomerase family protein
1809760 (+)	TTGTTCTGCATCAG	13.92 -9.54	PST_RS08530	41	DUF2059 domain-containing protein
1897290 (+)	TTGTCGTGCATCAG	13.98 -9.54	PST_RS08910	294	polysaccharide biosynthesis tyrosine autokinase
1987893 (+)	TTGACGCGGGTCAA	14.05 -9.54	PST_RS09335	24	alpha/beta hydrolase
2125060 (+)	TTGACGGGCGTCAA	14.19 -9.54	PST_RS09910	-77	coproporphyrinogen III oxidase
2219382 (+)	TTGCTCGGAATCAA	14.65 -9.54	PST_RS10345	-186	hypothetical protein
2438238 (+)	TTGATCGGAATCAA	15.21 -9.54	PST_RS11260	-86	Hsp20/alpha crystallin family protein
2507966 (+)	TTGTCCTGAATCAG	14.04 -9.54	*clpA* PST_RS11575	126	ATP-dependent Clp protease ATP-binding subunit ClpA
2804118 (+)	TTGACATCCATCAG	14.13 -9.54	PST_RS12960	165	flagellar hook-length control protein FliK
3360088 (+)	TTGATTCAAGTCAA	13.86 -9.54	*hmpA* PST_RS15650	-100	NO-inducible flavohemoprotein
3808733 (+)	TTGATCGCCATCAA	14.89 -9.54	PST_RS17540	-67	hypothetical protein
3820916 (+)	TTGACCGCAATCAA	15.01 -9.54	PST_RS17610	-102	CbbQ/NirQ/NorQ/GpvN family protein
3822709 (+)	TTGTTTCCGATCAA	14.33	*nirJ*	-110	heme d1 biosynthesis radic
				-9.54	PST_RS17625
