# Supplementary material for: The function of Anr in the differential effects of oxygen levels on biofilm development and nitrogenase performance in Pseudomonas stutzeri A1501
Source: PLoS One. 2025 Sep 24;20(9):e0333183. doi: 10.1371/journal.pone.0333183 (PMC12459779; doi:10.1371/journal.pone.0333183)
Supplement: S5 Fig — A) Anr protein model B) RpoS protein model, C) A protein-protein interaction using clusPro web site predicted 29 models out of which model 26 had the lowest kinetic energy calculated which was −1113.1. D) Illustrating the position of interacting molecules in the globular structure of protein. (PDF) [file pone.0333183.s005.pdf]

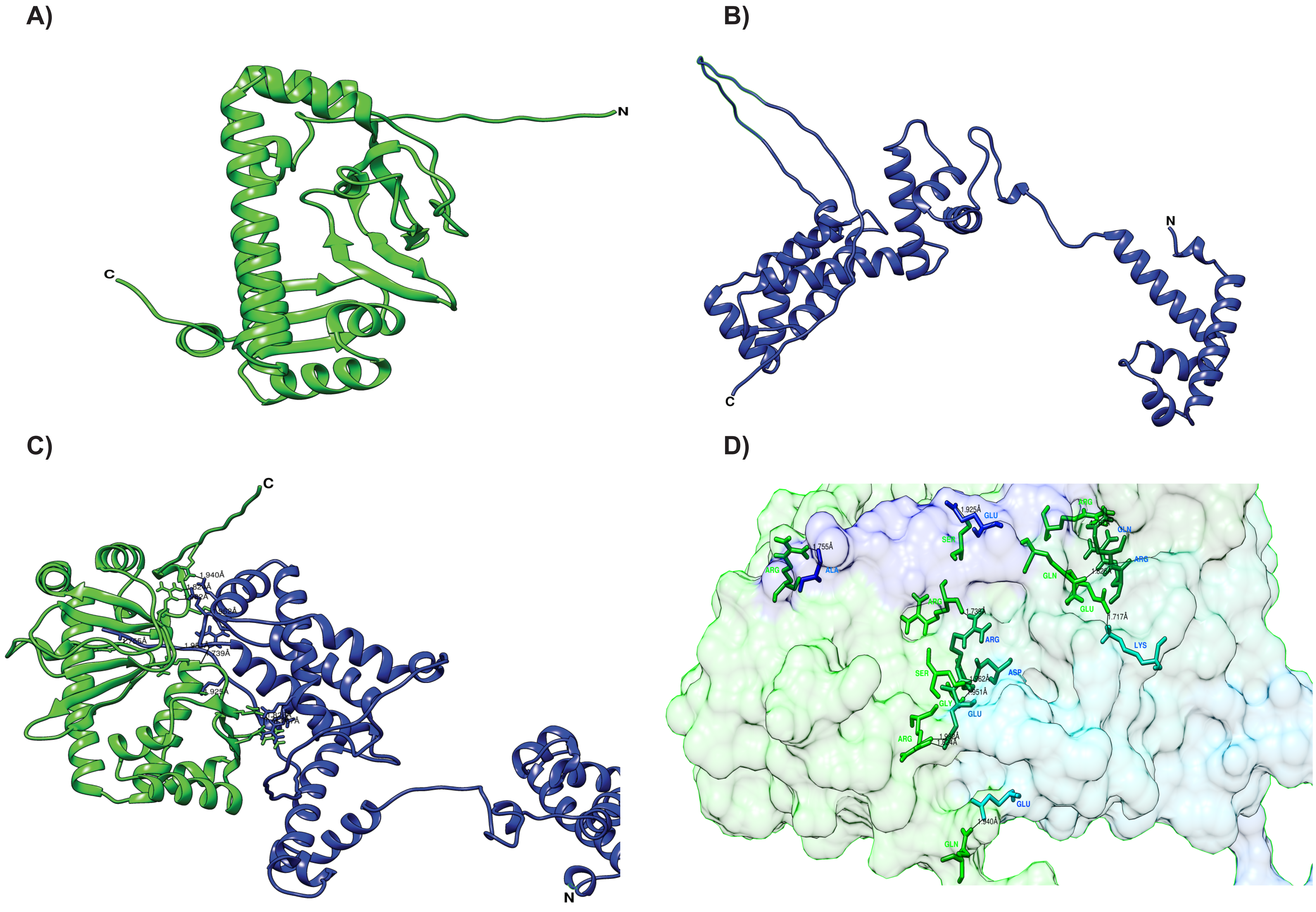

Supplementary Fig. S5. **Protein-Protein interaction** between RpoS and Anr protein using Chimera, and using the website (https://cluspro.org/login.php). **A)** Anr protein model **B)** RpoS protein model, **C)** A protein-protein interaction using clusPro web site predicted 29 models out of which model 26 had the lowest kinetic energy calculated which was -1113.1. **D)** Illustrating the position of interacting molecules in the globular structure of protein.
