# Supplementary material for: The function of Anr in the differential effects of oxygen levels on biofilm development and nitrogenase performance in Pseudomonas stutzeri A1501
Source: PLoS One. 2025 Sep 24;20(9):e0333183. doi: 10.1371/journal.pone.0333183 (PMC12459779; doi:10.1371/journal.pone.0333183)
Supplement: Attachment — Submitted filename: Response to Reviewers.docx [file pone.0333183.s012.docx]

PONE-D-25-31925

At low oxygen concentrations, the environmental impact of the anr gene on nitrogen fixation and biofilm formation in Pseudomonas stutzeri A1501

PLOS ONE

Dear Naga Raju,

Thank you for your email. I am also thankful for the reviewer’s insightful information on the manuscript. Please find the detailed point by point response on the article as below in blue,

1. Please ensure that your manuscript meets PLOS ONE's style requirements, including those for file naming. The PLOS ONE style templates can be found at

<https://journals.plos.org/plosone/s/file?id=wjVg/PLOSOne_formatting_sample_main_body.pdf> and

[https://journals.plos.org/plosone/s/file?id=ba62/PLOSOne_formatting_sample_title_authors_affiliations.pdf](https://track.editorialmanager.com/CL0/https:%2F%2Fjournals.plos.org%2Fplosone%2Fs%2Ffile%3Fid=ba62%2FPLOSOne_formatting_sample_title_authors_affiliations.pdf/1/010f0198c9e6ecec-36cc3b42-6e76-421d-aaf3-c2d3ca75b516-000000/ezsAsceYWXUp1S-2Psz00C2cln1-5rWdpHY1kVuWnw8=225).

**Answer:** In the new manuscript the style is strictly followed as per the PLOS one guideline available on the above-mentioned website.

2. PLOS ONE now requires that authors provide the original uncropped and unadjusted images underlying all blot or gel results reported in a submission’s figures or Supporting Information files. This policy and the journal’s other requirements for blot/gel reporting and figure preparation are described in detail at <https://journals.plos.org/plosone/s/figures#loc-blot-and-gel-reporting-requirements> and <https://journals.plos.org/plosone/s/figures#loc-preparing-figures-from-image-files>. When you submit your revised manuscript, please ensure that your figures adhere fully to these guidelines and provide the original underlying images for all blot or gel data reported in your submission. See the following link for instructions on providing the original image data: <https://journals.plos.org/plosone/s/figures#loc-original-images-for-blots-and-gels>.

In your cover letter, please note whether your blot/gel image data are in Supporting Information or posted at a public data repository, provide the repository URL if relevant, and provide specific details as to which raw blot/gel images, if any, are not available. Email us at [plosone@plos.org](mailto:plosone@plos.org) if you have any questions.

If there are ethical or legal restrictions on sharing a de-identified data set, please explain them in detail (e.g., data contain potentially sensitive information, data are owned by a third-party organization, etc.) and who has imposed them (e.g., an ethics committee). Please also provide contact information for a data access committee, ethics committee, or other institutional body to which data requests may be sent. If data are owned by a third party, please indicate how others may request data access.

**Answer:** In Fig-6 the entire Western blot for the Anr and RpoS has been added. The pictures are uncropped and in 300dpi TiFF format. Additionally, we shall send the All the protein blots by email to [plosone@plos.org](mailto:plosone@plos.org) for review.

3. Please remove your figures from within your manuscript file, leaving only the individual TIFF/EPS image files, uploaded separately. These will be automatically included in the reviewers’ PDF.

**Answer:** In Fig-6 the entire Western blot for the Anr and RpoS has been added. The pictures are uncropped and in 300dpi TIFF format. Additionally, we shall send the All the protein blots by email to [plosone@plos.org](mailto:plosone@plos.org) for review.

4. Please include captions for your Supporting Information files at the end of your manuscript, and update any in-text citations to match accordingly. Please see our Supporting Information guidelines for more information: <http://journals.plos.org/plosone/s/supporting-information>.

**Answer:** All supporting materials captions are mentioned in the end of the manuscript as per the guideline of PLOS one information provided by the official web site.

5. If the reviewer comments include a recommendation to cite specific previously published works, please review and evaluate these publications to determine whether they are relevant and should be cited. There is no requirement to cite these works unless the editor has indicated otherwise. 
**Answer:** The reviewer did not ask to add any previously published work in the review however; a previously published reference was added Reference number 26. It was presented in a seminar in 2019 at Shandong, China.

[Note: HTML markup is below. Please do not edit.]

Reviewers' comments:

Reviewer's Responses to Questions

**Comments to the Author**

1. Is the manuscript technically sound, and do the data support the conclusions?
The manuscript must describe a technically sound piece of scientific research with data that supports the conclusions. Experiments must have been conducted rigorously, with appropriate controls, replication, and sample sizes. The conclusions must be drawn appropriately based on the data presented.

Reviewer #1: Yes

Reviewer #2: Yes

2. Has the statistical analysis been performed appropriately and rigorously?

Reviewer #1: No

Reviewer #2: Yes

3. Have the authors made all data underlying the findings in their manuscript fully available?

The [PLOS Data policy](http://track.editorialmanager.com/CL0/http:%2F%2Fwww.plosone.org%2Fstatic%2Fpolicies.action%23sharing/1/010f0198c9e6ecec-36cc3b42-6e76-421d-aaf3-c2d3ca75b516-000000/ufQVPtD1zbWT5wQt8r7OhdhswD2Ige1g-sSUagLD3kc=225) requires authors to make all data underlying the findings described in their manuscript fully available without restriction, with rare exception (please refer to the Data Availability Statement in the manuscript PDF file). The data should be provided as part of the manuscript or its supporting information, or deposited to a public repository. For example, in addition to summary statistics, the data points behind means, medians and variance measures should be available. If there are restrictions on publicly sharing data—e.g. participant privacy or use of data from a third party—those must be specified.

Reviewer #1: Yes

Reviewer #2: Yes

4. Is the manuscript presented in an intelligible fashion and written in standard English?

PLOS ONE does not copyedit accepted manuscripts, so the language in submitted articles must be clear, correct, and unambiguous. Any typographical or grammatical errors should be corrected at revision, so please note any specific errors here.

Reviewer #1: No

Reviewer #2: No

5. Review Comments to the Author

Please use the space provided to explain your answers to the questions above. You may also include additional comments for the author, including concerns about dual publication, research ethics, or publication ethics. (Please upload your review as an attachment if it exceeds 20,000 characters)

Reviewer #1: General Comments:
1. Novelty and Significance:
The study provides valuable insights into the regulatory role of the *anr* gene in *P. stutzeri* A1501, especially its influence on nitrogen fixation and biofilm formation under microaerobic conditions. The connection between Anr, RpoS, and nitrogenase activity is novel and well explored.
2. Clarity and Structure:
The manuscript is rich in detail but suffers from redundancy and occasional repetition, particularly in the Results and Discussion sections. Some paragraphs would benefit from summarization for better flow and clarity.
3. Language and Grammar:
There are numerous grammatical issues and awkward phrasings throughout the manuscript. A professional language edit is recommended to improve readability and scientific clarity.

**Major Comments:**
¬ The abstract provides a solid summary, but it is too lengthy and technically dense. Simplifying and shortening would improve impact.

**Answer:** The abstract was not shortened as it might loss its importance to the study for the scientific audience. However small modification was done to make it more academic sound.

¬ The introduction is comprehensive, though some references (e.g., [1] to [7]) lack proper citation formatting. Consider shortening the background and focusing more clearly on the gap in knowledge addressed by this study.

**Answer:** The references were updated to have relevant formatting as per requirement of PLOS one. The introduction is concise without changing the core focus of the study to build the foundation of it.

¬ Experimental protocols are clearly described, but include strain source references directly in the tables instead of relying on supplementary figures alone. Better explain why certain oxygen concentrations (0.5%, 1.0%, 1.5%) were selected. The oxidative stress section lacks a positive control or a rationale for H₂O₂ dosage

**Answer:** Table (b) was added in the manuscript for explaining strains used in this study. The importance of oxygen in nitrogenase activity is explained from line 303 to 312. The different concentration shows that the oxygen *Pseudomonas stutzeri* A1501, oxygen concentration is not a simple. It is a key regulatory signal that determines the metabolic mode of the bacterium. Its ability to sense and respond to fine gradients of O₂ switching between aerobic respiration, denitrification, and nitrogen fixation is what makes it a highly successful and adaptable plant-associated bacterium in its natural environment. The rational for the dosage of the H_2_O_2_ is explained in Method and Material of Oxidative stress line number 195.

¬ While the results are rich in detail, they are narratively heavy. Breaking them into shorter paragraphs with subheadings would enhance readability. Figures mentioned (e.g., Fig 2, Fig 3, Fig 4A/B, Fig 5A/B) are not included in the main file. Ensure all figures are clearly labeled and discussed. Redundancy exists in the explanation of Anr’s regulatory role across nitrogen fixation and oxidative stress, these should be consolidated.

**Answer:** Results are now focused and more concise and be easy for audience to understand. For clarification Figures are cited as per lines they are in the manuscript.

| Figures | Line number in Manuscripts |
| --- | --- |
| Fig-1 | Line number 155 |
| Fig-2 | Line number 282 |
| Fig-3 | Line number 324 |
| Fig-4 | Line number 444 & 447 |
| Fig-5 | Line number 550 & 565 |
| Fig-6 | Line number 660 |

Anr does not have any particular role in oxidative stress however *anr* importance in nitrogen fixation condition is expressed manuscript Check from line 429 till 443. As oxygen is key player controlling regulation of both *nif* island genes and *anr* makes it relevant to study.

¬ The discussion often reiterates findings from the results rather than providing critical analysis. Strengthen the comparative analysis with previous studies, especially for Pseudomonas spp. and biofilm formation pathways.
Consider discussing the limitations of the study more explicitly and suggesting future directions.
**Answer:** Discussion is now primarily focused on the work done. Understanding the genetic foundations of biofilm formation, particularly the function of the *anr* gene in *Pseudomonas stutzeri* A1501, is essential for the advancement of innovative therapeutic strategies. The capacity of bacteria to form biofilms is significantly associated with the virulence of isolates from deep-seated illnesses, underscoring its essential role in pathogenesis of other pseudomonas species. Consequently, examining the regulatory mechanisms, including those regulated by the *anr* gene, that facilitate biofilm formation presents significant opportunities for the development of tailored antibiofilm therapeutics. This necessity propels research into fundamental mechanisms such as quorum sensing, a cell density-dependent communication mechanism that regulates biofilm formation and resistance, signifying a prospective target for anti-biofilm strategies.
¬ Western blot interpretation is sound, but quantification (e.g., densitometry values) is not clearly described. Bioinformatics docking results (Anr-RpoS interaction) are presented but need experimental validation or at least acknowledgment of limitations.
**Answer:** The manuscript provided an explanation of the western blot's density. Because of a lack of funds, the bioinformatics docking finding was not validated; this restriction was acknowledged in the publication and left for further research. Kindly check the line number 635.

Other Minor comments
♣ Define abbreviations (e.g., RpoS, qPCR) at first mention.

**Answer:** RpoS and qPCR are abbreviated at line number 49 and 43 respectively. Other Abbreviations are also clearly defined in the manuscript.
♣ Avoid subjective phrases like “fascinating area of study” and instead use objective scientific language.

**Answer:** All nonacademic statements were replaced with academic/scientific language from the manuscript.
♣ Use consistent tense throughout the manuscript—currently fluctuates between present and past.

**Answer:** The entire manuscript was checked for grammatical errors. Hopefully now the flow of the manuscript will be found constant.

Reviewer #2: The present work entitled “At low oxygen concentrations, the environmental impact of the *anr* gene on nitrogen fixation and biofilm formation in *Pseudomonas stutzeri* A1501” is significant work to undertake for research. The manuscript is clear and easy to understand. Even though, in the manuscript there are some minor mistakes as well as grammatical mistakes, hence the authors should be corrected and improve the language.
The precise comments specified below:
¬ The authors should be followed journal format throughout the manuscript.

**Answer:** The PLOS one format was strictly followed in the updated manuscript.
¬ Revise the complete manuscript by native English speaker.

**Answer:** The entire manuscript was checked for grammatical errors. Hopefully now the flow of the manuscript will be found constant.
¬ Line No:100- interaction should be interaction

**Answer:** Updated
¬ Line No:108 – Pseudomonas should be Pseudomonas

**Answer:** Updated
¬ Line No: 113 - Cellvibrio, Halomonas, and Rhodospirillum. Should be Cellvibrio, Halomonas, and Rhodospirillum.

**Answer:** Updated
¬ Tables – weight (g)- need not mention 5 g 10 g 10 g etc., should be 5 10 10 etc.,

**Answer:** Updated
¬ In text some places 30 degrees Celsius in some places 30 oC, it should be maintained uniform throughout the manuscript.

**Answer:** Updated, now single format is followed.
¬ Line No:115 & 116: The suspension was agitated at 220 rpm, adhering to the methodology established by [12]- incomplete sentence.

**Answer:** Updated in Method and material section line number 206 in the new manuscript.
¬ Line No: 179 & 180 - We repeated the identical We repeated the identical experiments to observe the biofilm. The biofilm was treated with the biofilm was treated with violet Repeated the sentence.

**Answer:** Updated
¬ Line No: 312 & 313- The observation establishes a correlation between the abundance of oxygen and an increase in ER expression. Why it’s in red colour?

**Answer:** Updated and removed. It was a wrong statement which was removed.

6. PLOS authors have the option to publish the peer review history of their article ([what does this mean?](https://track.editorialmanager.com/CL0/https:%2F%2Fjournals.plos.org%2Fplosone%2Fs%2Feditorial-and-peer-review-process%23loc-peer-review-history/1/010f0198c9e6ecec-36cc3b42-6e76-421d-aaf3-c2d3ca75b516-000000/XNBNH9aZM9Zh8-P9notYmO1QefKpPTIPK607zIEK4MI=225)). If published, this will include your full peer review and any attached files.


If you choose “no”, your identity will remain anonymous but your review may still be made public.


**Do you want your identity to be public for this peer review?** For information about this choice, including consent withdrawal, please see our [Privacy Policy](https://track.editorialmanager.com/CL0/https:%2F%2Fwww.plos.org%2Fprivacy-policy/1/010f0198c9e6ecec-36cc3b42-6e76-421d-aaf3-c2d3ca75b516-000000/7inBCH4J6FUhX-ZIBLagOuZtQd0nfGDtft-4jVdXnpo=225).

Reviewer #1: No

Reviewer #2: No

[NOTE: If reviewer comments were submitted as an attachment file, they will be attached to this email and accessible via the submission site. Please log into your account, locate the manuscript record, and check for the action link "View Attachments". If this link does not appear, there are no attachment files.]

While revising your submission, please upload your figure files to the Preflight Analysis and Conversion Engine (PACE) digital diagnostic tool, [https://pacev2.apexcovantage.com/](https://track.editorialmanager.com/CL0/https:%2F%2Fpacev2.apexcovantage.com%2F/1/010f0198c9e6ecec-36cc3b42-6e76-421d-aaf3-c2d3ca75b516-000000/xc9lxZtZ_bXU0Aah1pT34iDL50a75kOWvcv-9G03RNs=225). PACE helps ensure that figures meet PLOS requirements. To use PACE, you must first register as a user. Registration is free. Then, login and navigate to the UPLOAD tab, where you will find detailed instructions on how to use the tool. If you encounter any issues or have any questions when using PACE, please email PLOS at [figures@plos.org](mailto:figures@plos.org). Please note that Supporting Information files do not need this step.

**Answer:** Figures were modified using PACE digital diagnostic tool and were updated on the manuscript account along with that the figures were sent to PLOS using the [Figure@plos.org](mailto:Figure@plos.org). in Zip file downloaded from PACE.

Thank you so much again for your valuable insight on my manuscript. I deeply would like to thank the reviewer and Editor for the important points to improve the manuscript making the it more scientifically science for the audience.

Please do let me know if you any other quarry related to my manuscript and I shall reply it as early as possible to you.
